# Acupotomy for patients with trigger finger: A systematic review protocol

**DOI:** 10.1097/MD.0000000000017402

**Published:** 2019-10-18

**Authors:** Yan Jia, Zuyun Qiu, Xiaojie Sun, Yifeng Shen, Qiaoyin Zhou, Shiliang Li

**Affiliations:** a Department of Acupuncture-moxibustion, China-Japan Friendship Hospital; b Beijing University of Traditional Chinese Medicine, Beijing; c Chengdu University of Traditional Chinese Medicine, Chengdu, Sichuan; d Fujian University of Traditional Chinese Medicine, Fuzhou, Fujian, China.

**Keywords:** acupotomy, protocol, systematic review, trigger finger

## Abstract

**Background:**

Trigger finger is thought to be caused by aseptic inflammation of the A1 pulley and subsequent thickening and narrowing of the fibrous sheath. Acupotomy has been an important treatment for trigger finger. But an updated systematic review about this issue has not yet been released. This systematic review protocol is aimed at providing a higher quality method used to evaluate the efficacy and safety of acupotomy treatment for trigger finger.

**Methods:**

The following databases will be searched from the study inception to July 2019: the Cochrane Central Register of Controlled Trials (Cochrane Library), MEDLINE, EMBASE, PubMed, China National Knowledge Infrastructure, Wan-Fang Data, and Chinese Biomedical Literature Database. All English or Chinese randomized controlled trials related to acupotomy for trigger finger will be included. Two reviewers will independently perform the processes of study inclusion, data extraction, and quality assessment. The primary outcome will be assessed by improvement of the pain symptoms and finger activity. Secondary outcomes will be assessed through Safety assessment. Meta-analysis will be completed by RevMan V.5.3 software.

**Results:**

This systematic review will provide an assessment of the current state of acupotomy for trigger finger, aiming to show the efficacy and safety of treatment.

**Conclusion:**

This systematic review will re-evaluate a higher-quality systematic review to obtain a relatively convincing conclusion that finds acupotomy to be a better choice for trigger finger patients.

**PROSPERO registration number:**

CRD42018118663

## 1 Introduction

Trigger finger, also known as stenosing flexor tenosynovitis, is a mechanical problem. It is thought to be caused by aseptic inflammation of the A1 pulley and subsequent thickening and narrowing of the fibrous sheath. When the flexor tendon passes the tendon sheath at the metacarpal head, this abnormal structure can lead to restricted movement of the tendon. Trigger finger has been a common hand disease that often requires surgery, and it has numerous etiological factors as possible causes. The thumb, long finger, and ring finger are most commonly affected. The condition often occurs in manual laborers, such as tilers, fitters, and housewives. Primary trigger finger is more common in patients between the ages of 50 and 60 years, and it is more prevalent in women than men.^[1]^ The diagnosis is mainly based on clinical symptoms presenting during examination. The condition is characterized by pain, clicking, and loss of motion in the affected finger. Sometimes a pea-sized induration can be felt near the metacarpophalangeal joint where the popping is generated. There is no role for x-rays in diagnosis if patients have no experience of inflammatory disease or trauma.^[2]^

Trigger finger can be treated nonsurgically by using splinting and corticosteroid injections. Surgery is not the first option unless patients continue to be symptomatic after conservative treatment. Surgery treatment options most often include percutaneous A1 pulley release and open A1 pulley release.^[3,4]^ In addition, acupotomy therapy is commonly used in clinical treatment in China.^[5–7]^

Acupotomy treatment uses a needle knife as the main treatment tool, which has a needle body with a knife tip. It is a new procedure that combines traditional Chinese acupuncture treatment with modern surgical principles. Acupotomy theory supposes that disorders of soft tissue structures are the risk factors for chronic soft tissue injury, which includes adhesion, contracture, scar formation, and blockage.^[8]^ Acupotomy treatment for trigger finger has its unique advantages, such as a small wound size, high efficacy rate, and low recurrence rate. An increasing number of clinical studies have reported that acupotomy is effective for the treatment of trigger finger. It has been one of the most important treatments for trigger finger in China.

The mechanism of acupotomy therapy is not yet clear. It works effectively for chronic soft tissue injury by peeling the adhesion, releasing the contracture, and clearing the blockage.^[8]^ The mechanism to treat trigger finger using acupotomy is similar to percutaneous A1 pulley release. Some literature sources^[9–12]^ have reported that acupotomy can effectively release the thickened tendon sheath, relieve the pressure of the flexor tendon, and help the recovery of the normal metacarpophalangeal joint structure.

The applied value of acupotomy in treating trigger finger is obvious. However, the safety of this operation is often questioned owing to its closed surgery approach, which is mostly performed by relying on hand sensations. Tendons, blood vessels, and nerves may get damaged during treatment.^[13]^ Until now, only 1 systematic review of acupotomy for trigger finger has been published.^[14]^ The conclusion of literature published in 2016 only assessed acupotomy as being superior to steroid injection based on the efficacy rate, and there were no significant differences in adverse reactions. However, 3 years have passed since the publication of the 2016 evaluation, and many new experiments in the field have been published during this time.^[15–18]^ Ultrasound guidance technology is also gradually being applied.^[17,18]^ What's important is that our latest anatomy test has confirmed the efficacy and safety of acupotomy treatment. However, an updated systematic review or research program on this issue has not yet been released. Therefore, it is important to re-evaluate a higher-quality systematic review to obtain a relatively convincing conclusion that finds acupotomy to be a better choice for trigger finger patients.

## 2 Methods

### 2.1 Inclusion criteria for study selection

#### 2.1.1 Types of studies

Randomized controlled trials (RCTs) of acupotomy for patients with trigger finger will be included without restrictions on language and publication status. Nonrandomized studies will be excluded for further data syntheses while the data of the acupotomy group will be extracted for a safety assessment.

#### 2.1.2 Types of patients

Trials must include participants who meet diagnostic criteria.^[1,19,20]^ Participants with trigger finger, regardless of age, gender, race, educational status, and symptom stage, will be included. Trials involving study participants who are not eligible for acupotomy treatment owing to pre-existing conditions, such as fractures, dislocations, tumors, and other serious illnesses, will be excluded.

#### 2.1.3 Types of interventions

##### 2.1.3.1 Experimental interventions

The treatment group will receive acupotomy therapy without any limits to the needle shape, material, or treatment process.

##### 2.1.3.2 Control interventions

The control group without acupotomy interventions will receive either acupuncture, sham acupotomy, placebo control, steroid injection therapy, massage, or other conventional therapies. This review will also include an evaluation of acupotomy combined with another treatment modality and compared with the same treatment alone.^[21,22]^ Trials that compare different needle insertions or different forms of needle knives will be excluded.

#### 2.1.4 Types of outcome measures

##### 2.1.4.1 Primary outcomes

The primary outcome will be improvement of the pain symptoms and finger activity. This will be assessed through validated questionnaires such as Quinnell Grade 1,^[23]^ diagnostic efficacy of standard TCM syndrome,^[19]^ and visual analog scale. Trials with nonvalidated questionnaires or no clear descriptions of evaluation methods will be excluded.

##### 2.1.4.2 Secondary outcomes

Secondary outcomes will include a safety assessment, as judged by incidence rate and severity of adverse effects (eg, pain or limited activity), and a quality of life assessment based on the recurrence rate after at least 3 months of treatment.^[7]^

### 2.2 Search methods for the identification of studies

Regardless of the publication status, the following databases will be searched electronically from study inception to July 2019: the Cochrane Central Register of Controlled Trials (Cochrane Library), EMBASE, PubMed, MEDLINE, China National Knowledge Infrastructure, Wan-Fang Data, and Chinese Biomedical Literature Database. The databases will be searched for acupotomy RCTs of trigger finger. The search strategy will be created according to the Cochrane handbook guidelines. Search terms will be as follows: Acupotomy therapy, acupotomy, the needle knife, trigger finger, tenosynovitis of hand flexor tendons, flexor tendon stenosis tenosynovitis, flexor muscle tenosynovitis, randomized controlled trials, random trials, and clinical trials. The trials included will not be restricted by language and publication status. For Chinese databases, the search terms will be accurately translated. The search strategies for PubMed are summarized in Table 1.

**Table 1:**
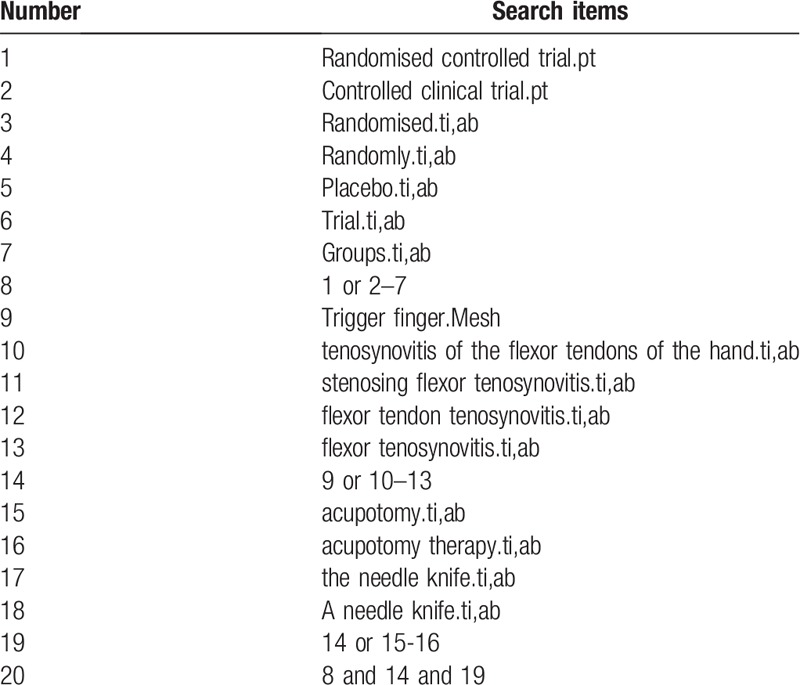
Search strategy used in PubMed.

#### 2.2.1 Searching other resources

We will search potential eligible trials through scanning the reference lists of identified publications. In addition, we will search other electronic sources, including the Cochrane Library and Turning Research Into Practice databases, for existing systematic reviews to select as relevant references to be cited in the study. Conference proceedings and unpublished literature will also be included.

### 2.3 Data collection and analysis

#### 2.3.1 Selection of studies

**Figure 1:**
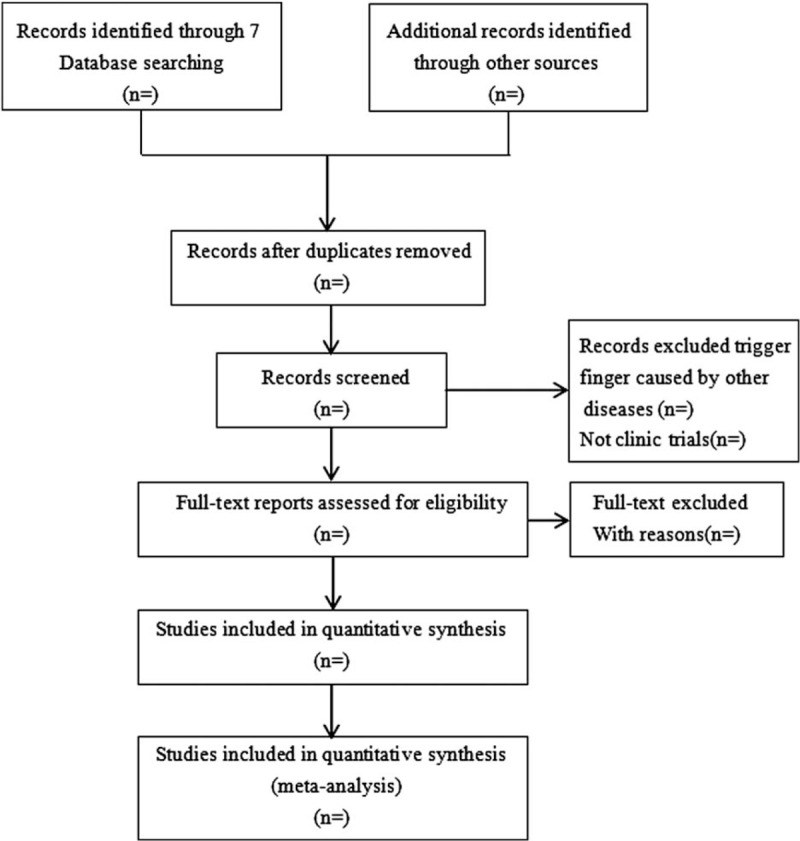
Flow diagram of the study selection process.

Each reviewer will receive training to ensure a full understanding of the purpose and process of the review. Reviewers will use Endnote X7 software to manage the trials that have been searched and remove duplicates. Two reviewers will independently review the titles, abstracts, and keywords of all the potentially eligible references to decide which trials will satisfy the inclusion criteria. The results of the selection process will be cross-checked by 2 reviewers. A trial will be excluded if both reviewers agree that it does not meet eligibility criteria. Any disagreements will be resolved with a senior reviewer through a group discussion. The selection procedure for the study is shown in Figure 1.

#### 2.3.2 Data extraction and management

The data extraction for the selected reports or studies will be completed by 2 authors independently via data forms. Any differences or uncertainties found during the check will be resolved through discussion and recommendations with a senior reviewer. The data extraction forms will include the following: author's information, time of publication, characteristics of participants, method of randomization, blinding, interventions, treatment course, evaluation index, outcomes, and adverse events. When the details are not granted explicitly in an article, the authors will be contacted.

#### 2.3.3 Assessment of risk of bias in included studies

Two reviewers will evaluate the risk of bias based on the Cochrane Collaboration's tool.^[24]^ We will evaluate the following 7 domains: random sequence generation, allocation hiding, blinding of participants, people and results, incomplete results data, selective reporting, and other biases. The risk of bias will be classified as low, unclear, and high. When the details are not granted explicitly in an article, the authors will be contacted.

#### 2.3.4 Measures of treatment effect

As for dichotomous data analysis, risk ratios with 95% confidence intervals (CIs) will be used. Mean difference with 95% CIs will be used for continuous data analysis. If scales are the same, weighted mean differences will be used for the data measured. Otherwise, standardized mean differences will be used.

#### 2.3.5 Dealing with missing data

We will try to request additional information from the corresponding authors of the original trials to provide missing or incomplete trial data if possible. If we are unable to obtain the missing data, a reliable analysis will be performed based on the available data and tested by a sensitive analysis. Otherwise, we will synthesize the rest of the available data. The possible impact of missing data will be discussed if necessary.

#### 2.3.6 Assessment of heterogeneity

A standard *χ*^2^ test will be completed for the detection of heterogeneity. The *I*^2^ statistic is going to be used to quantify inconsistencies among the studies. An *I*^2^ value of 50% or more will be considered to have substantial heterogeneity.

#### 2.3.7 Assessment of reporting bias

When the trials included are more than 10, funnel plots will be generated to detect the reporting bias. We will perform the Egger regression test to assess plots visually.

#### 2.3.8 Data synthesis

Data synthesis will be performed using RevMan V.5.3 software from the Cochrane Collaboration. Based on the results of *χ*^2^ and *I*^2^, we will choose to use either fixed-effects or random-effects models. The heterogeneity of each trial will not be considered if the *I*^2^ value is less than 50%, and the fixed effects model will be used for combined data. If substantial statistical heterogeneity is found, a random-effects model will be chosen. If significant clinical heterogeneity is found, we will perform a subgroup analysis, or analyze the characteristics and differences of the included studies.

#### 2.3.9 Subgroup analysis

To explain the heterogeneity, we will conduct a subgroup analysis depending on the specific situation. The premise is that we must get complete data. Factors such as subgroups of different acupotomy types and interventions will be taken into account.

#### 2.3.10 Sensitivity analysis

We will conduct a sensitivity analysis to guarantee the robustness of the review conclusions, methodological quality, sample size, and the effect of missing data. The studies of lower quality will be excluded. The analysis will then be discussed and repeated.

#### 2.3.11 Grading the quality of evidence

The quality of evidence for all outcomes will be judged through the grading of recommendations assessment, development, and evaluation. The assessments will be divided into 4 levels: high, moderate, low, or very low.^[25]^

## 3 Discussion

This systematic review will provide an assessment of the current state of acupotomy for trigger finger, aiming to show the efficacy and safety of treatment. The analysis of this systematic review will be divided into 4 parts: identification, study inclusion, data extraction, and data synthesis. Conclusions generated from this review may benefit clinicians, policymakers, and patients with trigger finger. On the other hand, there are some potential limitations that must be addressed in this review. The first concern is that high heterogeneity may arise from the various criteria for efficacy evaluation and different forms of acupotomy. Different operating methods may also cause this problem. The second issue is that the quality of included reports may be irregular, which will limit the ability to draw a conclusion based on high confidence. Presently, the effective treatment choices for patients with trigger finger are various; however, there is no clear and unified treatment protocol. Therefore, we will do our best to improve the methods of this comprehensive systematic review and draw convincing conclusions. Researchers and clinicians will formulate the most appropriate treatment plans for different degrees of patients based on these results.

## Author contributions

**Conceptualization:** Yan Jia, Zuyun Qiu.

**Data curation:** Yan Jia, Zuyun Qiu.

**Formal analysis:** Yan Jia, Zuyun Qiu, Yifeng Shen, Qiaoyin Zhou.

**Methodology:** Yan Jia, Zuyun Qiu.

**Project administration:** Yan Jia, Zuyun Qiu.

**Resources:** Yan Jia, Zuyun Qiu, Xiaojie Sun.

**Software:** Yan Jia, Zuyun Qiu, Yifeng Shen, Qiaoyin Zhou.

**Supervision:** Shiliang Li.

**Writing – original draft:** Yan Jia, Zuyun Qiu.

**Writing – review and editing:** Yan Jia, Zuyun Qiu, Shiliang Li.
